# Mitigation of greenhouse gas emissions through optimized irrigation and nitrogen fertilization in intensively managed wheat–maize production

**DOI:** 10.1038/s41598-020-62434-9

**Published:** 2020-04-03

**Authors:** Xin Zhang, Guangmin Xiao, Hu Li, Ligang Wang, Shuxia Wu, Wenliang Wu, Fanqiao Meng

**Affiliations:** 1 College of Resources and Environmental Sciences, Hebei Agricultural University, Baoding, 071000 China; 2 Beijing Key Laboratory of Farmland Soil Pollution Prevention and Remediation, Beijing Key Laboratory of Biodiversity and Organic Farming, College of Resources and Environmental Sciences, China Agricultural University, Beijing, 100193 China; 3 Institute of Agricultural Resources and Regional Planning, Chinese Academy of Agricultural Sciences, Beijing, 100081 China

**Keywords:** Environmental impact, Climate-change mitigation

## Abstract

In the wheat–maize rotation cultivation system in northern China, excessive irrigation and over-fertilization have depleted groundwater and increased nitrogen (N) losses. These problems can be addressed by optimized N fertilization and water-saving irrigation. We evaluated the effects of these practices on greenhouse gas emissions (GHG), net profit, and soil carbon (C) sequestration. We conducted a field experiment with flood irrigation (FN0, 0 kg N ha^−1^ yr^−1^, FN600, 600 kg N ha^−1^ yr^−1^) and drip fertigation treatments (DN0, 0 kg N ha^−1^ yr^−1^; DN420, 420 kg N ha^−1^ yr^−1^; DN600, 600 kg N ha^−1^ yr^−1^) in 2015–2017. Compared with FN600, DN600 decreased direct GHGs (N_2_O + CH_4_) emissions by 21%, and increased the net GHG balance, GHG intensity, irrigation water-use efficiency (IWUE), and soil organic C content (ΔSOC) by 13%, 12%, 88%, and 89.8%, respectively. Higher costs in DN600 (for electricity, labour, polyethylene) led to a 33.8% lower net profit than in FN600. Compared with FN600, DN420 reduced N and irrigation water by 30% and 46%, respectively, which increased partial factor productivity and IWUE (by 49% and 94%, respectively), but DN420 did not affect GHG mitigation or net profit. Because lower profit is the key factor limiting the technical extension of fertigation, financial subsidies should be made available for farmers to install fertigation technology.

## Introduction

Agricultural sustainability implies that high crop yields can be maintained under extreme natural conditions and that agricultural practices have acceptable environmental impacts^1^. Many countries including China will have to adopt appropriate intensive production practices to ensure the sustainability of agriculture^2^. Globally, agricultural soil is thought to be one of the major contributors to greenhouse gas (GHG) emissions, accounting for 10%–12% of total anthropogenic GHGs^3^. However, agricultural soil also sequesters 4 × 10^8^ − 9 × 10^8^ Mg C yr^−1^ ^4^.

Northern China, one of the most intensified agricultural regions, accounts for only 25% of the farmland in China, but produces about 66% and 28% of the country’s wheat (*Triticum aestivum* L.) and maize (*Zea mays* L.), respectively^5^. Currently, nitrogen (N) use by crops is still inefficient in this region, resulting in losses of up to 70% of applied fertilizer N^6^. If the agricultural N application rate (550–600 kg N ha) was reduced by 30%–60%, N losses may be decreased by more than 50%^7^. In Huantai County, northern China, agriculture is strongly dependent on pumped deep groundwater for irrigation, and the irrigation water-use efficiency (IWUE) is very low (<2.5 m^−3^ kg^−1^)^8^. The high annual depletion rate of groundwater (2.2 ± 0.3 cm) in this area is mainly due to the long-term overuse of water resources^9^. Since the 1990s, various sound farming options, e.g., (drip) fertigation, have been proposed and implemented to reduce the use of fertilizer N and irrigation water without decreasing the high crop yields in this region^10^.

Fertigation, where mineral fertilizer is supplied via a drip system, provides more appropriate amounts of nutrients (e.g., N) and water to the active plant root zone than does broadcast fertilization^11^. Drip fertigation has been proven to decrease methane (CH_4_) emissions and N losses (NH_3_, N_2_O, and NO) and increase the soil organic carbon (SOC) content^12^, while maintaining similar crop yields to those achieved under flood irrigation^13,14^. However, some studies have also found that fertigation with similar N levels to those of flood irrigation may increase GHGs emissions^11^. This is because denitrification is stimulated by the increase in water-filled pore spaces (WFPS) due to frequent irrigation^11^. Besides the effects on direct GHGs (N_2_O, and CH_4_), (drip) fertigation may also increase CO_2_ emissions because of increased energy consumption to run the system^15^ and to install the drip fertigation facilities^16^. In addition, large amounts of CO_2_ are produced during the manufacturing and transport of chemical fertilizers for crop production^16^. Compared with conventional flood irrigation, drip fertigation has different effects on the soil carbon (C) stock, because of differences in soil moisture content, temperature, and the initial decomposition rate of straw^12,17^. It is, therefore, critically important to comprehensively monitor and evaluate GHGs emissions, resource utilization, and land productivity of fertigation *vs*. flood irrigation, to optimize wheat/maize production in the intensified cropping region of northern China.

In view of the above, we established a field experiment in winter wheat–summer maize cropping system in northern China during 2015–2017, and monitored direct and indirect GHGs emissions, soil C sequestration, and production costs. The objectives of this study were to: (i) monitor the direct and indirect GHGs emissions and SOC stock changes with different irrigation and N fertilization combinations; (ii) assess the efficacy of optimized N fertilization and irrigation practices to mitigate GHGs emissions and maintain a high net profit; and (iii) provide suggestions and discuss the implications for sustainable agriculture in the future. On the basis of our results, we can propose strategies to improve the sustainability of agriculture in this region.

## Results

### Environmental conditions

**Figure 1:**
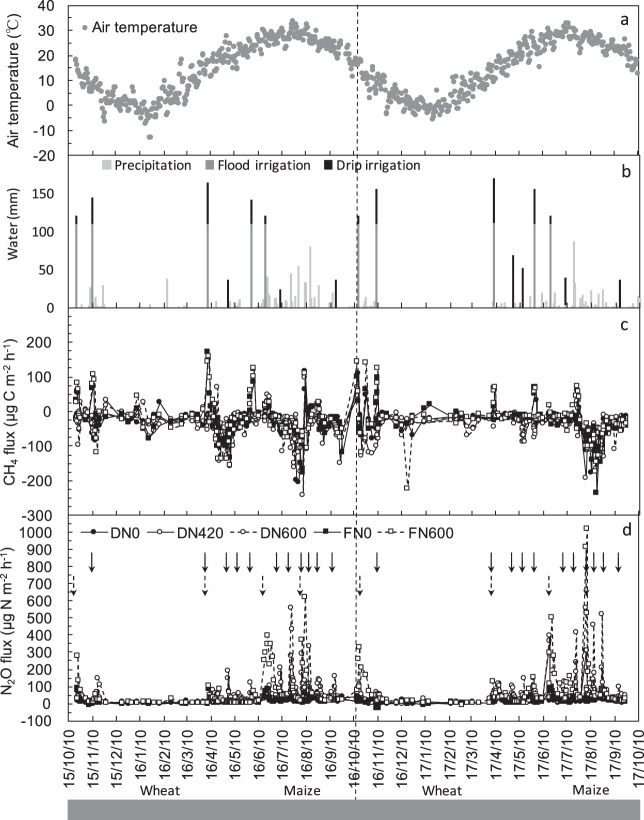
(**a**) Mean daily air temperature (°C), (**b**) water inputs (mm day^−1^) from precipitation (light gray bars) and irrigation (dark gray and black bars), (**c**) CH_4_ fluxes (μg C m^−2^ h^−1^), (**d**) N_2_O fluxes (μg N m^−2^ h^−1^) in 2015–2017 cropping years. Dotted arrows in (**d**) indicate N fertilizer application dates. Solid arrows and dash arrows in (**d**) denote time of N fertilization in fertigation and flood treatments, respectively. Definitions of different fertilizer management systems (i.e., FN0, DN0, FN600, DN420, and DN600) are provided in footnote of Table 1.

Annual precipitation was higher in the 2015–2016 cropping year than in the 2016–2017 cropping year (Fig. 1b). Correspondingly, less irrigation was applied in 2015–2016 than in 2016–2017 (Table 1). In 2015–2016, the seasonal irrigation in the drip fertigation treatments (DN600, DN420, and DN0) was 62.5% lower in the wheat season and 36.4% lower in the maize season, compared with those in the flood irrigation treatments (FN600 and FN0). Similarly, in 2016–2017, seasonal irrigation in the drip fertigation treatments was 36.8% lower in the wheat season and 26.4% lower in the maize season, compared with those in the flood irrigation treatments.

**Table 1:** N rate (kg N ha^−1^), irrigation (mm) and Rainfall (mm) during the two cropping years.

Crop	Stage	2015–2016	Date	Irrigation		Rainfall	2016–2017	Date	Irrigation		Rainfall
		N rate					N rate				
		DN0/DN420/DN600/FN0/FN600		Fertigation	Flood		DN0/DN420/DN600/FN0/FN600		Fertigation	Flood	
Wheat	Sowing	0/0/0/0/120	20/10/15	10	110	53.8	0/0/0/0/120	14/10/16	10	110	57.4
	Tillering	0/28.4/40.5/0/0	09/11/15	34	110	103.8	0/28.4/40.5/0/0	7/11/16	45	110	9.3
	Jointing	0/37.8/54/0/150	05/04/16	54	110	8.6	0/37.8/54/0/150	06/04/17	58	110	17.6
	Booting	0/47.3/67.5/0/0	30/04/16	36	0	39.9	0/47.3/67.5/0/0	30/04/17	68	0	7.4
	Flowering	0/47.3/67.5/0/0	15/05/16	0	0	25.5	0/47.3/67.5/0/0	13/05/17	51	0	23.1
	Filling	0/28.4/40.5/0/0	30/05/16	31	110	0	0/28.4/40.5/0/0	28/05/17	46	110	27.6
	Harvesting	—	15/06/16	—	—	—	—	15/06/17	—	—	—
	Sum	0/189/270/0/270		165	440	231.6	0/189/270/0/270		278	440	142.4
Maize	Sowing	0/0/0/0/120	17/06/16	10	110	109.3	0/0/0/0/120	17/06/17	10	110	30.9
	Jointing	0/34.7/49.5/0/0	07/07/16	24	0	71.9	0/34.7/49.5/0/0	07/07/17	35	0	127.2
	Small bell	0/34.7/49.5/0/0	21/07/16	0	0	69.9	0/34.7/49.5/0/0	21/07/17	0	0	49.4
	Large bell	0/46.2/66/0/210	04/08/16	0	0	65.2	0/46.2/66/0/210	03/08/17	0	0	25.3
	Tasseling	0/46.2/66/0/0	13/08/16	0	0	108	0/46.2/66/0/0	13/08/17	0	0	50.2
	Filling	0/46.2/66/0/0	24/08/16	0	0	56.5	0/46.2/66/0/0	24/08/17	0	0	16.5
	Ripening	0/23.1/33/0/0	15/09/16	36	0	0	0/23.1/33/0/0	15/09/17	36	0	39.4
	Harvesting	—	10/10/16	—	—		—	10/10/17	—	—	—
	Sum	0/231/330/0/330		70	110	480.8	0/231/330/0/330		81	110	338.9
Annual		0/420/600/0/600		235	550	712.4	0/420/600/0/600		359	550	481.3

Note: FN600: local farmers’ average level of N fertilizer application and flood irrigation; DN600: conventional level of N fertilizer application and drip fertigation; DN420: optimal level of N fertilizer application and drip fertigation; DN0: no N fertilizer applied and drip irrigation; and FN0: no N fertilizer applied and flood irrigation; Fertigation includes the treatments of DN0, DN420 and DN600; Flood irrigation includes the treatments of FN0 and FN600.

Because watering was more frequent in the drip fertigation treatments, the soil WFPS (0–10 cm depth) was higher in DN600 (65.2% ± 0.5%) than in the flood irrigation treatments (62.4% ± 1.3%) during the whole experiment. Consequently, soil temperatures (0–5 cm depth) were lower in the drip fertigation treatments (DN600, 17.8 ± 0.2 °C) than in the flood irrigation treatments (FN600, 19.2 ± 0.2 °C). The average WFPS of DN420, DN600, and FN600 was 64.9% ± 1.3%, 64.9% ± 1.3%, and 73.8% ± 1.1%, respectively, in the 2015–2016 cropping year, and 67.5% ± 1.2%, 68.9% ± 1.1%, and 80.2% ± 2.3%, respectively, in the 2016–2017 year, within 7 days after each irrigation event (Table S1).

### Crop yield and water-use and nutrient-use efficiencies

Compared with the local conventional flood irrigation and fertilization practice, i.e., the FN600 treatment, DN600 resulted in higher yields of winter wheat and summer maize in both the 2015–2016 and 2016–2017 growing seasons (Table 2). DN600 also increased the IWUE by 87.9%. However, the partial factor productivity for N fertilizer (PFP, an indicator for nitrogen-use efficiency) was not significantly different between DN600 and FN600 (Table 2).

**Table 2:** Yield (Mg ha^−1^), direct GHG (N_2_O, and CH_4_; Mg CO_2_-eq ha^−1^ yr^−1^), indirect GHG (fertilizer, power use for irrigation, fuel, pesticide, polyethylene lines (PE), seeds, and labor; Mg CO_2_-eq ha^−1^ yr^−1^), SOC sequestration rates (ΔSOC, Mg CO_2_-eq ha^−1^ yr^−1^) and their net GHG balance (Mg CO_2_-eq ha^−1^ yr^−1^), greenhouse gas intensity (GHGI, kg CO_2_-eq Mg^−1^ grain), PFP (kg kg^−1^ yr^−1^), and IWUE (kg m^−3^ yr^−1^) during the two cropping years (mean ± SE, *n* = 3).

	Yield	Direct GHG emission		Indirect GHG emission							ΔSOC^a^	Net GHG balance^b^	GHGI	PFP^c^	IWUE^d^
		N_2_O	CH_4_	Fertilizer	Electricity	Fuel	Pesticide	PE	Seed	Labor					
DN0^e^	12.0 ± 0.7b^f^	0.43 ± 0.03d	−0.11 ± 0.00b	0.61	5.80	1.91	0.04	1.46	0.21	0.32	1.81 ± 0.89a	8.86 ± 0.92ab	737 ± 58a		4.04 ± 0.23b
DN420	16.1 ± 1.1a	1.18 ± 0.10c	−0.13 ± 0.00c	4.10	5.80	2.66	0.04	1.46	0.21	0.32	3.97 ± 2.50a	11.7 ± 2.6ab	751 ± 220a	38.4 ± 2.5a^d^	5.44 ± 0.35a
DN600	15.6 ± 0.2a	1.61 ± 0.02b	−0.12 ± 0.01bc	5.59	5.80	2.20	0.04	1.46	0.21	0.32	3.17 ± 2.80a	13.9 ± 2.8a	891 ± 173a	26.0 ± 0.3b	5.26 ± 0.05a
FN0	9.13 ± 0.29c	0.50 ± 0.02d	−0.09 ± 0.00a	0.61	3.93	1.91	0.04	0	0.21	0.09	1.05 ± 0.35a	6.14 ± 0.34b	674 ± 44a		1.66 ± 0.05d
FN600	15.4 ± 0.5a	1.97 ± 0.08a	−0.09 ± 0.00a	5.59	3.93	2.20	0.04	0	0.21	0.09	1.67 ± 1.13a	12.3 ± 1.1ab	795 ± 60a	25.7 ± 0.9b	2.80 ± 0.10c

Note: ^a^ΔSOC denotes the changes in SOC density in the two-year rotation cycle, positive ΔSOC values indicate SOC accumulation in 0–30 cm soil layer;

^b^Net GHG balance and GHGI were calculated by Eqs. (6) and (7);

^c^PFP (Partial factor productivity for N fertilizer): grain yield/N fertilizer rate;

^d^IWUE (irrigation water use efficiency): grain yield/irrigation water rate;

^e^Definitions of the different fertilizer managements (i.e., FN0, DN0, FN600, DN420, and DN600) are referred to the footnotes of  Table 1;

^f^The same letter in the same column denotes no significant difference in different treatments by LSD (*P* < 0.05).

During the 2-year field study, the optimized irrigation and fertilization practice, i.e., DN420, resulted in the highest annual average grain yield of 16.1 Mg ha^−1^ (6.9 and 9.2 Mg ha^−1^ for wheat and maize, respectively), and significantly increased IWUE by 94% (*P* < 0.05) and PFP by 49% (*P* < 0.05), compared with their respective values in FN600 (Table 2). Compared with the DN600 treatment, the DN420 treatment increased IWUE by 3.4% and PFP by 47.7%.

### CH_4_ emissions

**Figure 2:**
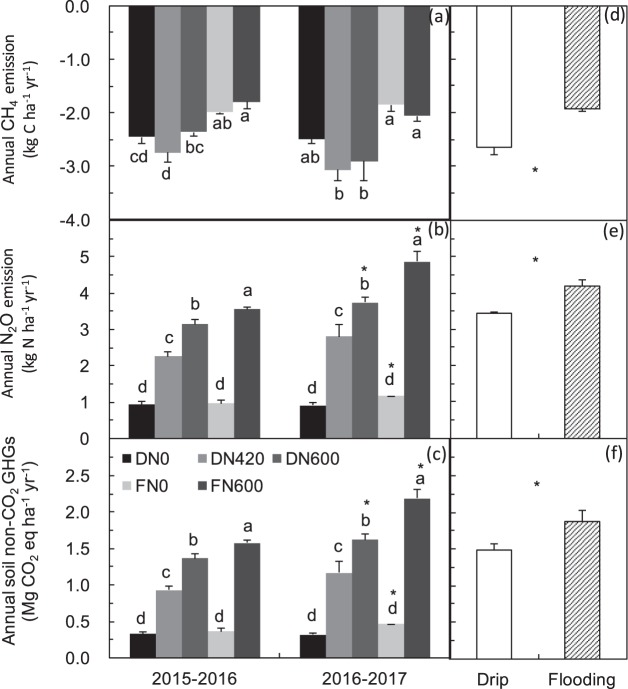
Annual N_2_O emission (kg N_2_O-N ha^−1^ yr^−1^), CH_4_ emission (kg CH_4_-C ha^−1^ yr^−1^), and total non-CO_2_ GHGs (N_2_O + CH_4_, Mg CO_2_-eq ha^−1^ yr^−1^) in 2015–2017 cropping years (**a–c**) and different irrigation treatments (at the same level of 600 kg N ha^−1^ yr^−1^, i.e., DN600 and FN600; **d–f**). Different letters denote significant differences among treatments (LSD test, *P* < 0.05); yearly differences in (**a–c**) and different irrigation treatments in (**d–f**) denoted by *P < 0.05. Negative annual CH_4_ flux values indicate CH_4_ uptake. Definitions of different fertilizer management systems (i.e., FN0, DN0, FN600, DN420, and DN600) are provided in footnote of Table 1.

The CH_4_ fluxes coincided well with irrigation and precipitation events (Fig. 1c). Of all the CH_4_ fluxes, 94.7% (drip) and 81.7% (flood) of the observations were negative fluxes, i.e., CH_4_ uptake or consumption. The maize season accounted for 40%–44% (2015–2016) and 36%–45% (2016–2017) of total annual CH_4_ uptake. Compared with the FN600 treatment, the DN600 treatment increased CH_4_ uptake by 31.8% (*P* < 0.05) in 2015–2016, and by 41.3% (*P* < 0.05) in 2016–2017 (Fig. 2a), a mean annual increase of 36.6% (Fig. 2d). Compared with the DN600 treatment, the DN420 treatment increased CH_4_ uptake by 16.5% in 2015–2016 (*P* < 0.05) and by 5.2% in 2016–2017.

### N_2_O emissions

In all N treatments, the N_2_O flux peaked within about 3 days after fertilizer application in the wheat growing season, but within 2 days after fertilizer application in the maize growing season. Fewer N_2_O flux events occurred from December to March, corresponding to the low soil temperature (−4.9–9.4 °C). The details of N_2_O fluxes in 2015–2016 have been described in our previous study. The maize season accounted for 63%–75% (2015–2016) and 63%–70% (2016–2017) of total annual N_2_O emissions (Fig. 1d). The seasonal cumulative N_2_O emissions in the FN600 treatment were 0.87 (wheat, 2015–2016), 2.67 (maize, 2016), 1.7 (wheat, 2016–2017), and 3.16 kg N_2_O-N ha^−1^ (maize, 2017). Compared with the FN600 treatment, the DN600 treatment decreased the wheat seasonal N_2_O emissions by 34.7% in 2016–2017 (*P* < 0.05) and by 16.1% in 2015–2016; and the DN600 treatment decreased the maize seasonal N_2_O emissions by 20.2% in 2016 (*P* < 0.05) and by 17.1% in 2017 (*P* < 0.05). Compared with the DN600 treatment, the DN420 treatment decreased the wheat seasonal N_2_O emissions by 21.8% (2015–2016) and 17.1% (2016–2017), and decreased the maize seasonal N_2_O emissions by 31.5% (2016) and 28.6% (2017) (Fig. 2).

### Direct and indirect GHGs, SOC stock changes, net GHG balance, and GHGI

The annual direct GHGs (N_2_O + CH_4_) emissions in the FN600 treatment were 1.88 Mg CO_2_-eq ha^−1^ yr^−1^, which was 26.2% higher than in DN600 (*P* < 0.05) and 44.1% higher than in DN420 (*P* < 0.05) (Fig. 2c,f). In contrast, the annual indirect GHGs emissions were 29.6% higher in DN600 than in FN600 (12.1 Mg CO_2_-eq ha^−1^ yr^−1^), mainly because of the higher electricity consumption and polyethylene required for irrigation facilities in DN600 (Table 2). Because DN420 received 30% less N fertilizer than did DN600, its indirect GHGs emissions were 6.4% lower than those of DN600. Direct + indirect GHGs emissions were 23% and 9% higher in DN600 (17.1 Mg CO_2_-eq ha^−1^ yr^−1^) than in FN600 and DN420, respectively. These results show that, in the life-cycle of wheat and maize production, indirect GHGs are main contributors to overall climate effects.

After the 2-year experiment, the SOC content (0–30 cm) in DN420 and DN600 had increased by 0.52 and 0.41 g kg^−1^, respectively, significantly more than in FN600 (0.22 g kg^−1^, *p* < 0.05) (Table 2). That is, the increase in SOC stock (0–30 cm) for FN600 in the 2-year experimental period was 0.46 Mg C ha^−1^ yr^−1^, significantly lower than that in DN420 (1.08 Mg C ha^−1^ yr^−1^) and DN600 (0.86 Mg C ha^−1^ yr^−1^).

The annual net GHG balance values were greater than zero for all management systems, indicating that all systems were net GHGs sources (Table 2). Over the 2-year period, the total net GHG balance was 13.0% higher in DN600 (13.9 Mg CO_2_-eq ha^−1^ yr^−1^) than in FN600. However, the total net GHG balance of DN420 was 15.8% and 4.9% lower than those of DN600 and FN600, respectively.

The net GHG balance per unit grain yield is usually defined as GHGI. The GHGI and yield in DN600 (891 kg CO_2_-eq Mg^−1^ grain) were 12.1% and 1.3% higher, respectively, than those in FN600. The GHGI and yield in DN420 were 15.7% lower and 3.2% higher, respectively, than those in DN600.

## Discussion

Various factors affect GHGs emissions under field production, including the type and quantity of fertilizer applied^18^, irrigation^19^, previous crop residue management^20–22^, soil properties, and climatic factors^23^.

In agricultural ecosystems such as cropland, fertilizer N may promote nitrification and denitrification by nitrifiers to produce N_2_O, but inhibit the enzyme responsible for CH_4_ oxidation, thus increasing annual CH_4_ emissions^3,4^. However, lower levels of N fertilization (<100 kg ha^−1^) tend to stimulate methanotrophy^24,25^ and decrease N_2_O production^26,27^. At each drip fertigation event, the urea was directly applied to the crop rhizosphere with water at a greater frequency (five times for wheat and six times for maize) but at a lower rate (<70 kg N ha^−1^) than in the FN600 treatment (twice for wheat and maize, respectively, 120–210 kg N ha^−1^). This may have caused the higher CH_4_ uptake in DN600 and DN420 than in FN600. As discussed in our previous study, within 7 days after fertilization, the soil inorganic N pool was significantly higher in flood-irrigated soils (91% higher in the wheat season and 55% higher in the maize season) than in drip-irrigated soils^17^. This increased the N-use efficiency, which would have decreased the level of soil inorganic N (NO_3_^−^-N + NH_4_^+^-N) available for transformation into N_2_O by microorganisms in the drip fertigation system^13^, as proven in previous studies^11,28^. As discussed in our previous study, in addition to nitrification oxidation, nitrifier denitrification also substantially contributes to the generation and diffusion of N_2_O, and after oxidation of NH_4_^+^ to NO_2_^−^, more N is transformed into N_2_O and NO than into NO_3_^−17^. In the present study, the soil NO_3_^−^ content during the 2-year period was 24% lower in FN600 (40 mg kg^−1^) than in DN600 (53 mg kg^−1^), indicating that more soil NO_2_^−^ was transformed into N_2_O or NO via nitrifier denitrification in FN600 than in DN600. This has been discussed in our previous paper^17^.

The optimum WFPS range for N_2_O emission is 70%–80%^14^. The production of N_2_O via denitrification occurs at WFPS > 70%, whereas N_2_O is produced via nitrification at WFPS < 70%^29^. CH_4_ can be produced only in strictly anaerobic conditions^30^. A lower WFPS (e.g., DN600 *vs*. FN600: 67% *vs*. 77% within 7 days after each irrigation event during the 2-year study) has been shown to increase soil aeration, promote NH_2_OH oxidation, increase the conversion of N_2_O into NO^31^, and increase CH_4_ oxidation^30^. Additionally, N_2_O and CH_4_ emissions are known to be strongly and positively correlated with soil temperature^4^. Thus, the higher temperature in FN600 than in DN600 (19.2 °C *vs*. 17.8 °C) promoted N_2_O and CH_4_ production^17^.

Optimum farming systems may result in greater soil C sequestration as SOC^4,15^, thus contributing to greater CH_4_ uptake and reduced N_2_O emission^32^. As Lal^33^ reported, the rate of SOC accrual was only 0.3–0.5 Mg C ha^−1^ yr^−1^ under intensive agricultural practices, but was able to reach 1.0–1.5 Mg C ha^−1^ yr^−1^ in irrigated farming if sufficient straw was incorporated into soil. In this study, the rate of increase in SOC content was much higher in DN600 (0.45 g kg^−1^ yr^−1^) than in FN600 (0.20 g kg^−1^ yr^−1^). There are a number of factors that may have contributed to the high rate of SOC increase in DN600. Firstly, fertigation in DN600 may have contributed to greater root biomass, thereby strengthening the major pathway of C input into soil^12^. Secondly, a large amount of crop residues was returned to the soil in DN600. Crop residues are a key factor in determining changes in SOC stocks^16^. Here, more organic materials (straw) were returned to soil in DN600 (wheat: 2032 kg C ha^−1^, maize: 2875 kg C ha^−1^) than in FN600 (wheat: 1878 kg C ha^−1^, maize: 2768 kg C ha^−1^). Thirdly, the initial SOC content in our study was lower than those reported for European and US cropland soils (14.5–23.2 g kg^−1^)^15,16^. Finally, the duration of our study (2 years) was shorter than those of other field studies (5–15 years)^34^.

Analyses of the overall effects of agricultural practices on climate should include measurements of direct and indirect GHGs emissions and SOC changes^15,16^. The ΔSOC and electricity consumption for irrigation were the main reasons for the differences in indirect GHGs emissions between drip and flood irrigation treatments (Table 2), similar to the results of Tellez-Rio *et al*.^3^. Compared with the net GHG balance in FN600, that in DN600 was higher and that in DN420 was lower. This highlighted that the higher SOC sequestration rate in the optimal fertigation (DN420) treatment significantly alleviated the higher direct + indirect GHGs emissions. Thus, DN420 was the best practice in terms of GHGs emissions and C sequestration, while DN600 showed the worst performance because the indirect GHGs emissions were higher than those in FN600. However, there was no significant benefit for GHG mitigation in DN420 because of the higher indirect GHGs emissions related to polythene and electricity consumption for irrigation. Therefore, some measures (e.g., reduced fertilizer N and electric power consumption) to mitigate indirect GHGs emissions should be developed to promote the extension of fertigation in northern China.

**Figure 3:**
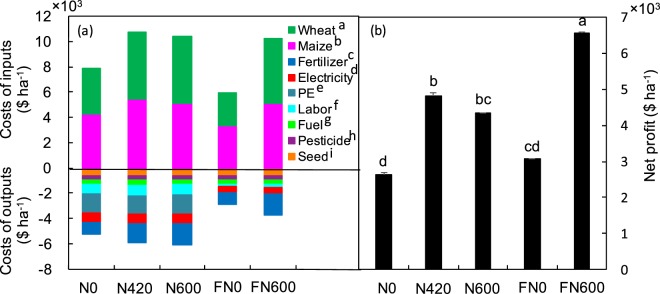
(**a**) Inputs ($ ha^−1^) and outputs ($ ha^−1^) and (**b**) net profit ($ ha^−1^) during the two cropping years (mean ± SE, *n* = 3). Definitions of different fertilizer management systems (i.e., FN0, DN0, FN600, DN420, and DN600) are provided in footnote of Table 1. Different letters in (**a**) denote significant difference among different treatments (LSD, *P* < 0.05). ^a,b^Prices for wheat and maize grain were $ 385.9 Mg^−1^ and $ 292.2 Mg^−1^, respectively (available at: www.grain.gov.cn); ^c^Prices for urea (46% of N), calcium superphosphate (17% of P_2_O_5_) and potassium sulphate (48% of K_2_O) were $ 282 Mg^−1^, $ 152 Mg^−1^, $ 519 Mg^−1^, respectively (available at: www.fert.cn); ^d–f^Prices for electricity, polyethylene lines, and labor were $ 0.085 KWh^−1^, $ 0.063 m^−1^ and $ ^1^.09 person^−1^ h^−1^, respectively (available at: www.sdwj.gov.cn); ^g^Price for diesel was $ 1.09 L^−1^ (available at: Youjia.ChemCp.com); ^h,i^Prices for pesticide, wheat (Luyuan 502) seed, and maize (Zhengdan 958) seed were $ 28.13 kg^−1^, $ 1.56 kg^−1^, and $ 3.75 kg^−1^, respectively (available at: www.sdny.gov.cn); net profit was defined as costs of outputs minus costs of inputs.

Currently, fertigation is used more for the production of vegetables and other cash crops than for cereal crops^10^ in northern China, because the economic output of vegetables (>$22000 ha^−1^) is much higher than that of wheat and maize (FN600: $6559 ha^−1^, Table S2). In this study, the net profit of FN600 was 51.1% and 35.9% higher than those of DN600 and DN420, respectively, due to the lower farming inputs in FN600 (i.e., lower power use, lower labour costs, and no polyethylene costs) (Fig. 3). The lower profit from crops cultivated using fertigation systems is the main factor restricting their use in northern China. In addition, the irrigation water is free and farmers have always over-irrigated croplands^10^. This further limits the use of fertigation, and has resulted in the formation of the largest groundwater funnel area worldwide, as reported by Feng *et al*.^9^. If a cost is introduced for irrigation water, then farmers will use it much more sparingly. This would reduce the depletion of groundwater and decrease soil N losses and groundwater pollution caused by excessive irrigation^8^, and raise farmers’ awareness of water saving as well.

In this study, a significant proportion of indirect GHGs emissions was associated with the use of electric power for irrigation. The inputs of electric power were 4463 and 3025 kwh ha^−1^ yr^−1^ for fertigation and flood irrigation, respectively; thus, electric power accounted for 33%–38% of total indirect GHGs emissions. If solar energy were recommended and if rainwater were stored for irrigation of crops, the consumption of electric power and underground water would be reduced. This would directly affect the GHG mitigation potential of the agricultural system.

The goal of the Chinese government is to achieve >3 million ha of farmland under fertigation by 2020 (Ministry of Agriculture and Rural Affairs of China, 2016). In Huantai County, the fertigation area was 1300 ha in 2017, with financial subsidies of up to 900 CNY per hectare. Our study highlights that other policies and financial schemes should be adopted to promote the environmentally friendly practice of optimized fertigation in China. For instance, in the 1990s, a pioneering financial subsidy policy in Huantai County helped farmers and cooperatives to purchase agricultural machinery for water-saving irrigation. The reduction in the amount of N fertilizer applied not only reduced the direct GHGs emissions from the soil, but also reduced the indirect GHGs emissions from production and transport, and reduced the input costs. For intensive agriculture in a semi-arid area, crops are very dependent on irrigation and chemical fertilizer (especially fertilizer N). Meanwhile, saving irrigation water combined with optimized fertilizer use is proven to be the key to decreasing underground water usage and soil N losses, while increasing crop yields and water- and N-use efficiencies^7,17,35^. In addition, financial subsidies and fertigation technologies may contribute to lower indirect GHGs emissions and production costs. An agricultural system based on fertigation represents an environmentally friendly system that can respond to the needs of food security and sustainable agriculture. Financial aid is a win–win solution to solve the problems of high production costs and indirect GHGs emissions associated with fertigation. Compared with conventional agriculture, this type of agricultural system will be better suited to respond to global climate change, and to safeguard food security in China.

## Conclusions

We evaluated different systems combining drip or flood irrigation with synthetic N fertilizer for winter wheat and summer maize production in northern China. The different systems resulted in differences in crop productivity, N-use and water-use efficiencies, GHGs emissions, and SOC stock enhancement. Compared with the flood irrigation treatment, optimized drip fertigation (DN420) maintained a lower WFPS and soil temperature, and a higher soil SOC content throughout the whole experimental period. Compared with the local conventional fertilization and flood irrigation treatments, the DN420 treatment also resulted in higher crop yield, IWUE, and PFP. However, compared with the flood irrigation system, the fertigation system had no significant benefit in terms of GHGs mitigation, because of higher indirect GHGs emissions (associated with polyethylene and electric power). The higher inputs of electric power, polyethylene, and labour led to 26.4%–33.8% lower net profits in the fertigation system than in the FN600 treatment. Consequently, the extension of fertigation technology is limited in northern China. For future sustainable and environmentally friendly agriculture, optimized drip fertigation is highly recommended in the intensively farmed wheat and maize production regions of northern China. Policies and financial programs are required to support the installation of fertigation technology. The overall aim is to ensure that farmers can afford to implement an agricultural system that can respond to global climate change, and safeguard food security.

## Materials and Methods

### Study area

The field experiment was conducted at Huantai Experimental Station, China Agricultural University, Shandong province (36°51′50″–37°06′00″N, 117°50′00″–118°10′40″E)^17^. The main cropping pattern in Huantai is the annual double cropping of winter wheat (*T. aestivum* L.) and summer maize (*Z. mays* L.). The region has a typical monsoon climate, with annual mean air temperature and precipitation of 12.5 °C and 542.8 mm, respectively. The experiment was conducted from Oct. 2015 to Oct. 2017 (2 years with four cropping seasons, Fig. 1a,b). The cumulative rainfall and mean air temperature during the 2 years were 712.4 mm and 14.7 °C, and 481.3 mm and 14.8 °C, respectively. The soil is a calcareous fluvo-aquic soil with a bulk density (0–30 cm) of 1.40 g cm^−3^, pH (1:2.5, soil/water) of 7.8, SOC content of 17.3 g kg^−1^, and total N content of 1.1 g kg^−1^. Since the 1980s, land productivity in this region has increased significantly with high rates of fertilizer application, frequent irrigation, and the cultivation of high-yielding varieties.

### Cropping systems

A completely randomized design was arranged with five treatments and three replicates, giving a total of 15 plots (each 10-m long and 5-m wide). The five treatments represented five different types of water and fertilizer N management, as follows: (1) local farmers’ conventional level of N fertilizer and flood irrigation (FN600, 600 kg N ha^−1^ yr^−1^); (2) local farmers’ conventional level of N fertilizer and drip fertigation (DN600, 600 kg N ha^−1^ yr^−1^); (3) optimal level of N fertilizer and drip fertigation (DN420, 420 kg N ha^−1^ yr^−1^); (4) no N fertilizer and drip irrigation (DN0, 0 kg N ha^−1^ yr^−1^); and (5) no N fertilizer and flood irrigation (FN0, 0 kg N ha^−1^ yr^−1^). Straw from the previous maize crop was mechanically chopped into pieces (5–8 cm length) and then incorporated into the field. Wheat (Luyuan 502) seeds were sown (row spacing, 40 cm) in mid-October and mature plants were harvested in early June of the following year. The wheat straw was mechanically chopped (2–5 cm length) and then incorporated into the field. Maize (Zhengdan 958) seeds were sown (row spacing, 60 cm) in mid- to late June and mature plants were harvested at the end of September.

For fertigation treatments, fields were irrigated by a surface drip irrigation system, with one line in each row of wheat/maize. Pressure-compensating emitters were spaced 30 cm apart. In the winter wheat and summer maize seasons, N fertilizer (urea, 46% N) and irrigation events were as described in the Supplementary Materials. Potassium sulphate (52% K_2_O) was applied as basal fertilizer at 118.3 kg K_2_O ha^−1^ for wheat and 84.7 kg K_2_O ha^−1^ for maize. Super phosphate (16% P_2_O_5_) was applied as basal fertilizer at 84.7 kg P_2_O_5_ ha^−1^ for wheat and 189 kg P_2_O_5_ ha^−1^ for maize. Table 1 lists the irrigation/fertilization times and amounts in all treatments.

### Measurement of GHG fluxes

From Oct 20, 2015 to Oct 10, 2017, N_2_O and CH_4_ fluxes were collected *in situ* simultaneously using a closed chamber method. The chamber was inserted into the soil (20 cm) under an emitter of one irrigation line in each experimental plot. During the sampling day, five air samples were taken at 8-min intervals while the chamber was closed using 35-ml polypropylene syringes and then stored in glass vials (30 ml). Gas was sampled from 8:00 to 11:00 local time in the morning for a continuous duration of 7–14 days following fertilization, rainfall, tillage and irrigation events, and twice per week during other periods. The N_2_O and CH_4_ levels in the samples were analysed with an Agilent 7820A gas chromatograph (Agilent, Santa Clara, CA, USA) within 24 h of sampling. The GHG fluxes were calculated from the five gas concentrations by nonlinear or linear methods, as described by Wang *et al*.^36^.

For indirect GHGs, chemical fertilizer (kg N ha^−1^; kg P_2_O_5_ ha^−1^, kg K_2_O ha^−1^) rates were calculated according to the experiment design. Power use for irrigation (electricity, Kw h ha^−1^) was calculated as follows:

1 $$Electricity\,(Kw\,h\,h{a}^{-1})=P\times \frac{I}{D}$$

where P is the power consumed by the water pump (Kw), I is the irrigation amount (m^3^ ha^−1^), and D is the water yield (m^3^ h^−1^). Fuel (kg ha^−1^) was the total diesel used for sowing and harvesting. Pesticides (kg ha^−1^), polyethylene lines in drip fertigation (kg ha^−1^), seeds (of wheat and maize; kg ha^−1^), and labour (day person^−1^ ha^−1^) were recorded for each event.

### Auxiliary measurements and carbon storage

Daily precipitation was recorded at a meteorological station located at the experimental station. The amount of irrigation water used in each event was recorded. During gas sampling, air temperature and topsoil (0–5 cm) temperatures were measured with a digital thermometer (JM624, JinMing Instrument Co. Ltd., Tianjin, China).

To determine WFPS and soil inorganic N (NO_3_^−^ − N and NH_4_^+^ − N) content, topsoil (0–10 cm) samples were collected at the same time that air samples were collected. Topsoil samples were collected from two different locations: under the dripper and from mid-way between adjacent drippers. This sampling method accurately represents water distribution around the crop plant for soil moisture detection^37^. The proportion of WFPS (%) was calculated using Eq. (2)^17^:

2 $$WFPS\,( \% )=\frac{Gravimetric\,water\,content\,( \% )\times Soil\,bulk\,density}{({1}-Soil\,bulk\,density/{2.65})}$$

where gravimetric water content was measured by drying the subsamples at 105 °C for 24 h, and 2.65 = theoretical particle density of soil (g cm^−3^).

The NH_4_^+^-N and NO_3_^−^-N contents of extracts were analysed using a continuous flow analyser (TRAACS2000, Bran and Luebbe, Norderstedt, Germany) after extraction with 1 M KCl (soil: solution = 1:5).

Here, we used soil organic carbon (SOC) content to represent C sequestered by soil. To determine the changes in SOC after the 2-year experimental period, composite soil samples were collected from each plot using a steel cylinder (5 cm diameter) at depths of 0–10, 10–20, and 20–30 cm. Soil samples were collected once (October) in 2015, twice (June and October) in 2016, and twice (June and October) in 2017, and air-dried and sieved through 0.5 mm mesh. The SOC content was determined with a CN analyser (Vario Max CN, Elementar, Hanau, Germany) after immersion of soil in 0.3 mol L^−1^ HCl solution for 24 h to remove carbonates and oven-drying at 65 °C. Bulk density (g cm^−3^) was measured using the conventional core method. The annual topsoil (0–30 cm) SOC sequestration rate (ΔSOC, kg CO_2_-eq ha^−1^ yr^−1^) was estimated from the change in topsoil SOC density (dSOC/dt, g C kg^−1^ yr^−1^) using Eq. (3)^16^:

3 $$\Delta SOC=dSOC/dt\times \rho \times {30}{/}{10}\times {44}{/}{12}$$

where, ρ is the bulk density (g m^−3^) of 0–30 cm depth topsoil; and 12 and 44 are the molecular weights of C and CO_2_, respectively.

### Calculation of partial productivity factor, IWUE, net GHG balance, and GHG intensity

The partial factor productivity for N fertilizer (PFP, kg kg^−1^) was defined as the ratio of crop yield to mineral fertilizer N applied^10^. The IWUE (kg m^−3^) was calculated as the ratio of grain yield (Y_g_, kg ha^−1^) to the total irrigation water consumed (W_g_, m^3^), as follows^17^:

4 $$PFP={Y}_{g}/{N}_{fert}$$

5 $$IWUE={Y}_{g}/{W}_{g}$$

where *Y*_*g*_ and *N*_*fert*_ are the grain yield (kg ha^−1^) and mineral fertilizer N rate (kg N ha^−1^), respectively.

Net GHG balance (kg CO_2_-eq ha^−1^) includes: (1) direct GHGs (soil N_2_O and CH_4_ emissions), and (2) indirect GHGs, i.e., upstream CO_2_ emissions from agricultural inputs (fertilizer, pesticides, seeds, power use for irrigation, fuel, polyethylene lines, and labour), and (3) SOC stock changes (0–30 cm layer) during the experimental period^30^. Hence, the net GHG balance and GHG intensity (GHGI) were calculated as follows^16^:

6 $$Net\,GHG\,balance\,(kg\,C{O}_{2}-eq\,h{a}^{-1}y{r}^{-1})=GW{P}_{Direct}+GW{P}_{Indirect}-\Delta SOC$$

Where GWP_Direct_ (kg CO_2_-eq ha^−1^) = GWP_N2O_ + GWP_CH4_, and represents the total CO_2_ emission equivalalents for soil N_2_O and CH_4_; GWP_Indirect_ (kg CO_2_-eq ha^−1^) = GWP_Fertilizer_ + GWP_Electricity_ + GWP_Fuel_ + GWP_Pesticide_ + GWP_PE_ + GWP_Seed_ + GWP_Labor_, and represents the total CO_2_ emission equivalalents for chemical fertilizer (N, P_2_O_5_, K_2_O) inputs, power use for irrigation, fuel, pesticides, polyethylene lines in drip fertigation, seeds (of wheat and maize), and labour, respectively (Table 3).

**Table 3:** Emission factors of N_2_O/CH_4_ emission and agricultural inputs.

Source	Abbreviation	Unit	Coefficients (kg CO_2_-eq unit^−1^)
N_2_O	*GWP*_*N*2*O*_	*kg N*_*2*_*O-N ha*^*−1*^	298
CH_4_	*GWP*_*CH*4_	*kg CH*_*4*_*-C ha*^*−1*^	34
Fertilizer	*GWP*_*N*_	*kg N ha*^*−1*^	8.3
	*GWP*_*P*2*O*5_	*kg P*_2_*O*_*5*_ *ha*^*−1*^	1.5
	*GWP*_*K2O*_	*kg K*_*2*_*O ha*^*−1*^	0.98
Power use for irrigation	*GWP*_*Electricity*_	*kW h ha*^*−1*^	1.30
Diesel fuel	*GWP*_*Fuel*_	*L ha*^*−1*^	3.93
Pesticide	*GWP*_*Pesticide*_	*kg ha*^*−1*^	18.0
Ppolyethylene lines	*GWP*_*PE*_	*kg ha*^*−1*^	8.7
Seeds	*GWPmaize*	*kg ha*^*−1*^	1.22
	*GWPwheat*	*kg ha*^*−1*^	1.16
Labor	*GWP*_*labor*_	*day person*^*−1*^ *ha*^*−1*^	0.86

Note: these data was cited from Gao, *et al*.^16^.

The GHGI (kg CO_2_-eq Mg^−1^ grain yield) represents the net GHG balance for production of a unit weight (Mg) of grain^15^, and was calculated as follows:

7 $$GHGI=Net\,GHG\,balance/grain\,yield$$

### Statistical analysis

All data were analysed using SPSS Statistics 22.0 software (SPSS Inc., Chicago, IL, USA) and plotted with Microsoft Excel 2016. Statistically significant differences were detected using the least significant differences (LSD) method (*P* < 0.05) in one-way analysis of variance (ANOVA). Differences were considered significant at *P* < 0.05.

## Supplementary information

Supplementary Information.
