# Supplementary material for: Mitigation of greenhouse gas emissions through optimized irrigation and nitrogen fertilization in intensively managed wheat–maize production
Source: Sci Rep. 2020 Apr 3;10:5907. doi: 10.1038/s41598-020-62434-9 (PMC7125187; doi:10.1038/s41598-020-62434-9)
Supplement: Supplementary file 1 — Supplementary Information. [file 41598_2020_62434_MOESM1_ESM.pdf]

Balancing greenhouse gas mitigation through optimized irrigation and N fertilization in intensively managed wheat-maize production

Journal name: *Scientific Reports*

Xin Zhang[1, 2+], Guangmin Xiao[2+], Hu Li[3], Ligang Wang[3], Shuxia Wu[3], Wenliang Wu[2], Fanqiao Meng[2*]

1 College of Resources and Environmental Sciences, Hebei Agricultural University, Baoding 071000, China

2 Beijing Key Laboratory of Farmland Soil Pollution Prevention and Remediation, Beijing Key Laboratory of Biodiversity and Organic Farming, College of Resources and Environmental Sciences, China Agricultural University, Beijing 100193, China

3 Institute of Agricultural Resources and Regional Planning, Chinese Academy of Agricultural Sciences, Beijing 100081, China

* Correspondence author. Email: mengfq@cau.edu.cn; Tell: 0086 10 62731538

+ These authors contributed equally to this work

* Correspondence author. Email: mengfq@cau.edu.cn

Contents: 7 pages, 1 Figures, 2 Tables.

Supporting Materials and Methods

Fertilization and irrigation events. In the winter wheat and summer maize seasons, N fertilizer (urea, 46% N) was applied at the ratio of 9:11 (wheat: maize), and details was shown in Table 1. Potassium sulfate (52% K_2O) was applied as basal fertilizers at 118.3 kg K_2O ha^{-1} for wheat and 84.7 kg K_2O ha^{-1} for maize, and super phosphate (16% P_2O_5) was applied as basal fertilizers at 84.7 kg P_2O_5 ha^{-1} for wheat and 189 kg P_2O_5 ha^{-1} for maize. For drip fertigations, the proportion of fertilizer-N in total fertilizer N at each growth stage was 0% (basal), 15% (tillering), 20% (jointing), 25% (booting), 25% (flowering), and 15% (grain filling) during the wheat season, and 0% (basal), 15% (jointing), 15% (small bell), 20% (large bell), 20% (tasseling), 20% (grain filling), and 10% (ripening) during the maize season.

Conventional irrigation treatments (FN600 and FN0) were flood irrigated depending on climatic conditions. For the drip irrigation treatments of DN600, DN420, and DN0, fields were irrigated by a surface drip irrigation system consisting of 12 pressure-compensated drip irrigation lines for wheat and 8 for maize per plot (one drip irrigation line for each line of wheat/maize), located on the soil surface. Pressure-compensating emitters were spaced 30 cm apart and provided water at a rate of 0.15 L h^{-1}. One fertilizer tank, sand filter, screen filter

and pressure gauge were contained in a fertigation plot. This system met the requirements of separated irrigation and fertilization with a total pressure in 0.3 Mpa in each district. The amount of drip water was calculated using Eq. (1)[1]:

$$Q=10\times H\times\left(\theta_{fc}-\theta_{0}\right) \quad (1)$$

where, H=depth of soil layer for calculating irrigation water (0-40 cm), cm; θ_{fc}=field water holding capacity; and θ_0=moisture content of soil layer (0-40 cm), %. The lowest irrigation limit in the current study was 85% of field water holding capacity.

Measurement of GHG fluxes. From Oct 20, 2015 to Oct 10, 2017, N_2O and CH_4 fluxes were measured *in situ* simultaneously using the closed chamber method[2]. Each chamber consisted of a square stainless-steel frame with a cross-sectional area of 0.25 m^2. The chamber was inserted into the soil to a depth of 20 cm in each experimental plot and kept in place throughout the entire study period, except when it was temporarily removed for necessary field operations such as tillage. On each sampling day, gases were sampled from 8:00 to 11:00 local time in the morning within 7–14 d after fertilization, rainfall, tillage and irrigation events. Sampling was conducted twice per week during other periods.

Before air sampling, chambers (base area of 0.25 m^2 and a height ranging from 0.5 to 1.5 m depending on crop size) were mounted onto the base frames and sealed with a rubber strip and clamps. During closure of the chambers, five air samples for GHG detection were taken at 8-min intervals using 35 ml polypropylene syringes and then stored in glass vials (30 ml).

Samples were analyzed using an Agilent 7820A gas chromatograph (Agilent, Santa Clara, CA, USA) equipped with an electron capture detector to analyze N_2O and a flame ionization detector to analyze CH_4. The GHGs fluxes were calculated from the five gas concentrations by nonlinear or linear methods, as described in detail by Wang *et al.*[2].

Table S1 WFPS (%) during 7 days after each irrigation events in DN420, DN600 and FN600

Treatment	DN420			DN600			FN600		
Period	2015-2016	2016-2017	2015-2017	2015-2016	2016-2017	2015-2017	2015-2016	2016-2017	2015-2017
WFPS	64.9±1.3	67.5±1.2	66.2±0.9	64.9±1.3	68.9±1.1	66.9±0.9	73.8±1.1	80.2±2.3	77.4±1.4

Note: FN600: local farmers' average level of N fertilizer application and flood irrigation; DN600: conventional level of N fertilizer application and drip fertigation; DN420: optimal level of N fertilizer application and drip fertigation

Table S2 Inputs (thousand \$ ha^{-1}) and outputs (thousand \$ ha^{-1}) and net profit (thousand \$ ha^{-1}) during the two cropping years (mean ± SE, n = 3)

	Inputs								Outputs			Net profit
	Fertilizer[a]	Electricity[b]	Fuel[c]	Pesticide[d]	PE[b]	Seed[d]	Labor[b]	SUM	Wheat[e]	Maize[e]	SUM	
DN0[f]	0.93	0.76	0.27	0.33	1.50	0.65	0.82	5.26	3.61	4.28	7.89	2.63±0.05d[g]
DN420	1.45	0.76	0.38	0.33	1.50	0.65	0.82	5.89	5.28	5.44	10.72	4.83±0.07b
DN600	1.68	0.76	0.31	0.33	1.50	0.65	0.82	6.05	5.26	5.14	10.4	4.35±0.01bc
FN0	0.93	0.52	0.27	0.33	0.00	0.65	0.22	2.92	2.65	3.33	5.98	3.06±0.02cd
FN600	1.68	0.52	0.31	0.33	0.00	0.65	0.22	3.71	5.19	5.07	10.26	6.56±0.04a

[a] Price for urea (46% of N), calcium superphosphate (17% of P_2O_5) and potassium sulphate (48% of K_2O) were \$ 282 Mg^{-1}, \$ 152 Mg^{-1}, \$ 519 Mg^{-1}, respectively (available at: www.fert.cn);

[b] Price for electricity, polyethylene lines and labor were \$ 0.085 KWh^{-1}, \$ 0.063 m^{-1} and \$ 1.09 $person^{-1}$ h^{-1}, respectively (available at: www.sdwj.gov.cn);

[c] Price for diesel was \$ 1.09 L^{-1} (available at: Youjia.ChemCp.com);

[d] Price for pesticide, herbicide, wheat (Luyuan 502) and maize (Zhengdan 958) seed were \$ 28.13 kg^{-1}, \$ 7.81 kg^{-1}, \$ 1.56 CNY kg^{-1} and \$ 3.75 CNY kg^{-1}, respectively (available at: www.sdny.gov.cn);

[e] Price for wheat and maize grain were \$ 385.9 Mg^{-1} and \$ 292.2 CNY Mg^{-1}, respectively (available at: www.grain.gov.cn);

[f] FN600: local farmers' average level of N fertilizer application and flood irrigation; DN600: conventional level of N fertilizer application and drip fertigation; DN420: optimal level of N fertilizer application and drip fertigation; DN0: no N fertilizer applied and drip irrigation; and FN0: no N fertilizer applied and flood irrigation;

[g] The same letter in the same column denotes no significant difference in different treatments by LSD ($P<0.05$).

References

1. Zhang, X. *et al.* Optimized fertigation maintains high yield and mitigates N_2O and NO emissions in an intensified wheat–maize cropping system. *Agr. Water Manage.* **211,** 26-36 (2019).
2. Wang, K. *et al.* Comparison between static chamber and tunable diode laser-based eddy covariance techniques for measuring nitrous oxide fluxes from a cotton field. *Agr. Forest Meteorol.* **171-172,** 9-19 (2013).
3. Grassini, P. & Cassman, K. G. High-yield maize with large net energy yield and small global warming intensity. *Proc. Natl. Acad. Sci. U. S. A.* **109,** 1074-1079 (2012).
4. Gao, B. *et al.* The impact of alternative cropping systems on global warming potential, grain yield and groundwater use. *Agrc. Ecosyst. Environ.* **203,** 46-54 (2015).
5. Wang, W. *et al.* Greenhouse gas intensity of three main crops and implications for low-carbon agriculture in China. *Climatic Change* **128,** 57-70 (2015).
